# An Unusual Case of Long-Segment Dissection of the Left Anterior Descending Coronary Artery During Osteoproximal Chronic Total Occlusion Intervention

**DOI:** 10.7759/cureus.46526

**Published:** 2023-10-05

**Authors:** Dibyasundar Mahanta, Deepak Kumar Parhi, Shilpa Vinayak Gadade, Debasish Das

**Affiliations:** 1 Department of Cardiology, Sunshine Hospital, Bhubaneswar, IND; 2 Department of Cardiology, SUM Hospital, Bhubaneswar, IND; 3 Department of Cardiology, All India Institute of Medical Sciences, Bhubaneswar, Bhubaneswar, IND

**Keywords:** left anterior descending coronary artery, osteoproximal, chronic total occlusion, coronary artery, dissection

## Abstract

Coronary artery dissection can occur after post-balloon inflation; however, a very long-segment coronary dissection (>50 mm) is a rare occurrence during routine coronary interventions. Here, we report an extremely rare case of long-segment coronary dissection in the left anterior descending coronary artery (LAD) induced during antegrade revascularization of chronic total occlusion of osteoproximal LAD with stiffer Gaia II wire. The patient had excruciating angina with hemodynamic collapse and acute pulmonary edema; the patient was rescued with long-segment coronary revascularization.

## Introduction

Coronary artery dissections are defined as the breach in the intimal layer of the coronary artery which can be either spontaneous or iatrogenic. The most common cause of iatrogenic coronary artery dissection is semi-compliant balloon inflation for lesion preparation during coronary angioplasty. During lesion preparation, the semi-compliant balloon fractures the calcium and dissects the atherosclerotic plaque which facilitates subsequent coronary angioplasty. Few dissections become a catastrophe during coronary intervention by producing abrupt vessel closure during which the patient becomes hemodynamically unstable, develops malignant ventricular arrhythmias, develops complete heart block, or develops acute pulmonary edema, and, rarely, may develop cardiac arrest. Long-segment coronary dissections rarely occur during coronary intervention [1] and most often occur during chronic total occlusion intervention. Long-segment coronary dissections are a nightmare for interventional cardiologists as the patient becomes highly symptomatic due to abrupt vessel closure with hemodynamic decompensation with acute pulmonary edema with desaturation [1]. Long-segment coronary dissections can be intraluminal, extraluminal, or intraluminal and extraluminal. During antegrade revascularization, high antegrade force to negotiate the wire forward creates a dissection which propagates further with further entry of the wire. The most interesting aspect of the present case is that it was a long-segment dissection of the coronary artery from osteoproximal to the distal segment, and the dissection traversed from the true lumen to the false lumen and again to the true lumen facilitating the distal passage of the wire. Dense calcium inside the wall of the coronary artery and artery tortuosity are predictors of difficult wire passage with a risk of dissection. Large and dense deposits of calcium inside the coronary artery obstruct the forward passage of the wire and make the wire penetrate the softer wall producing coronary dissection. The elderly, patients with a long history of chronic stable angina, and those with chronic kidney disease are the populations more likely to harbor dense coronary calcification with a risk of difficult intervention. The present case describes the rarest possibility of very long-segment coronary dissection during chronic total occlusion intervention which needs prompt revascularization with drug-eluting stents as patients with long-segment coronary dissection are more likely to be hemodynamically unstable with desaturation due to abrupt vessel closure.

## Case presentation

**Figure 1:**
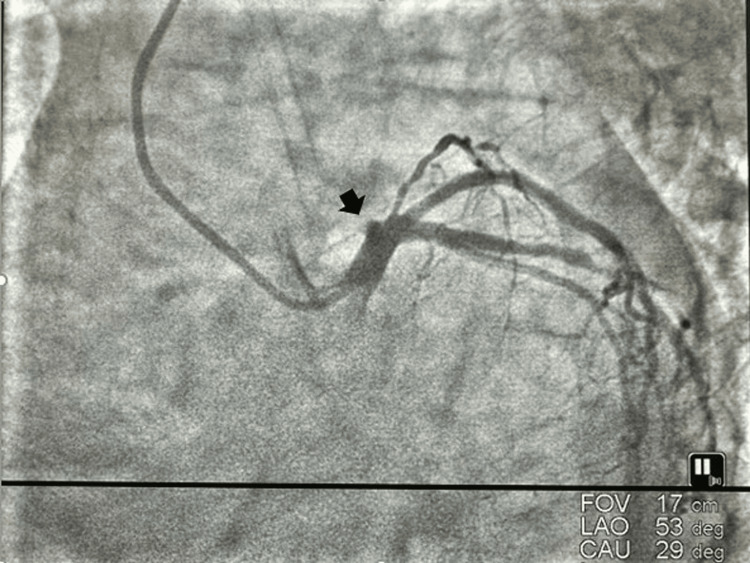
Chronic total occlusion of osteoproximal left anterior descending coronary artery.

**Figure 2:**
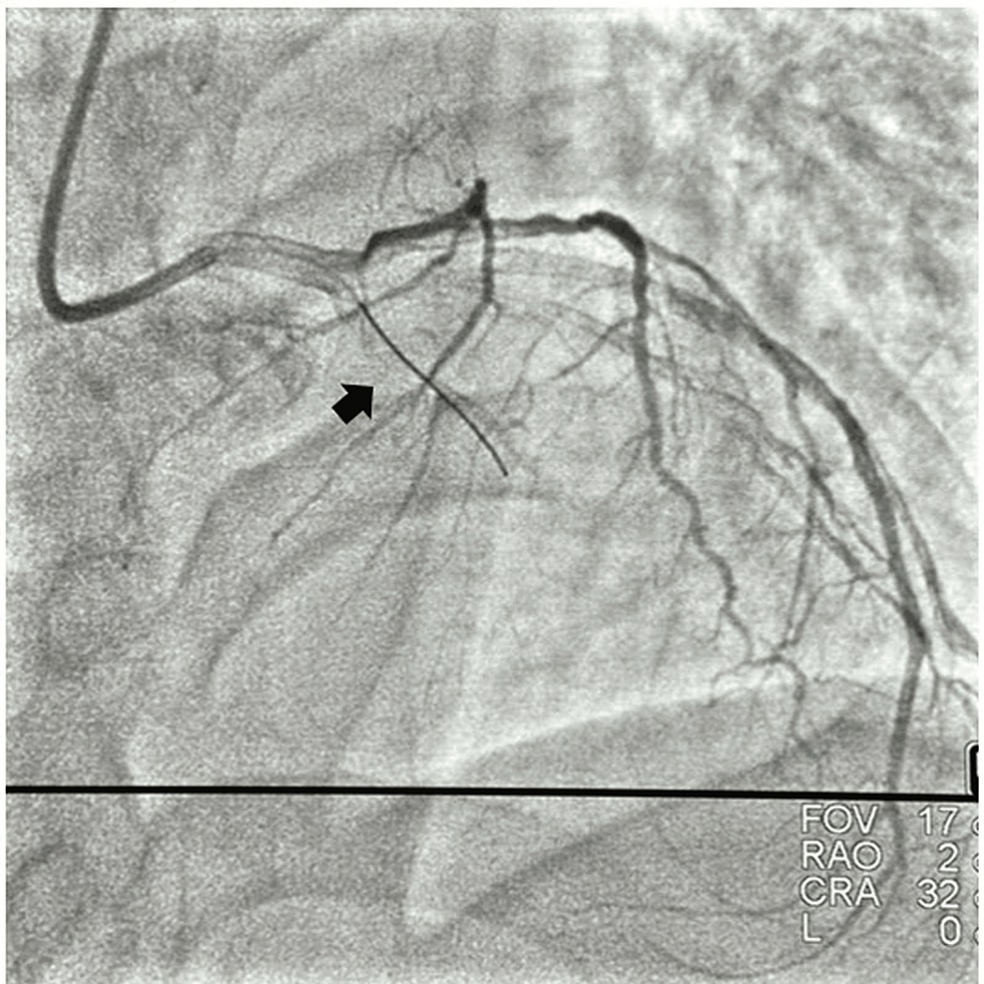
Antegrade wire in the false lumen.

**Figure 3:**
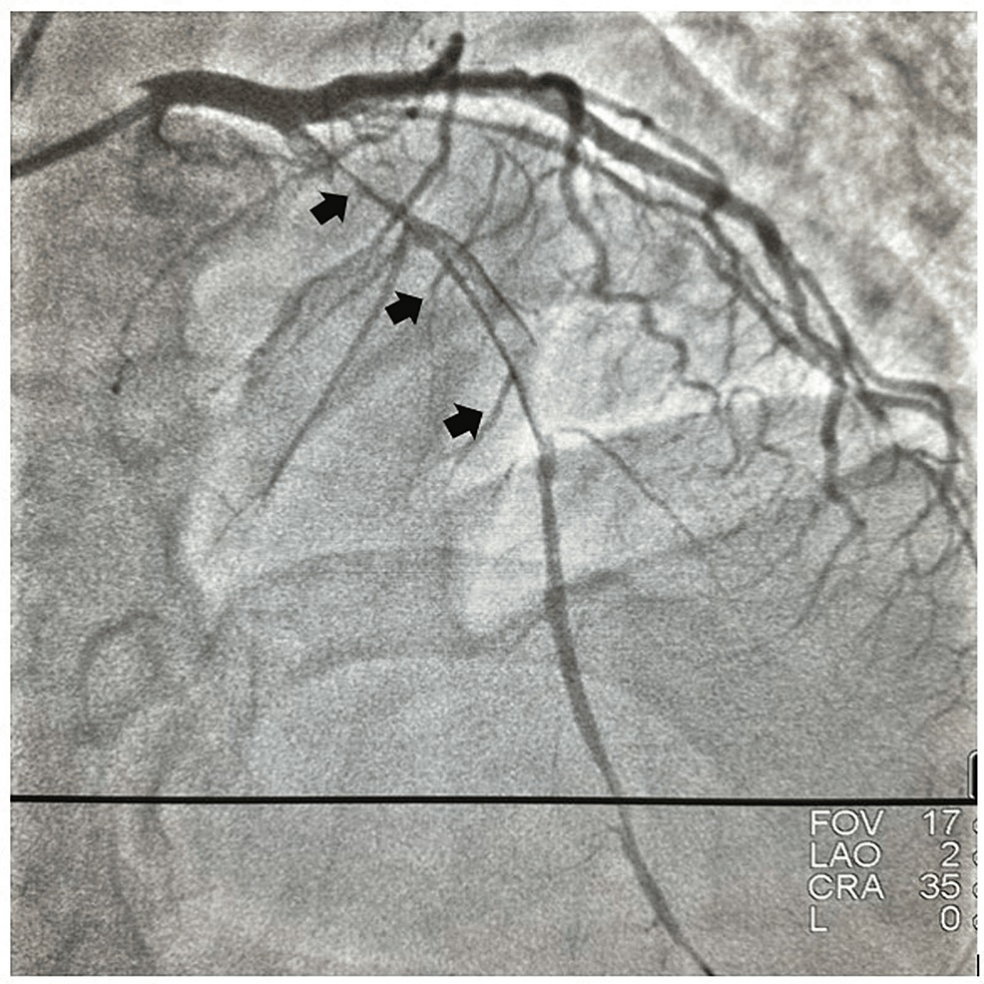
Long-segment dissection from true lumen-false lumen-true lumen in the left anterior descending coronary artery.

**Figure 4:**
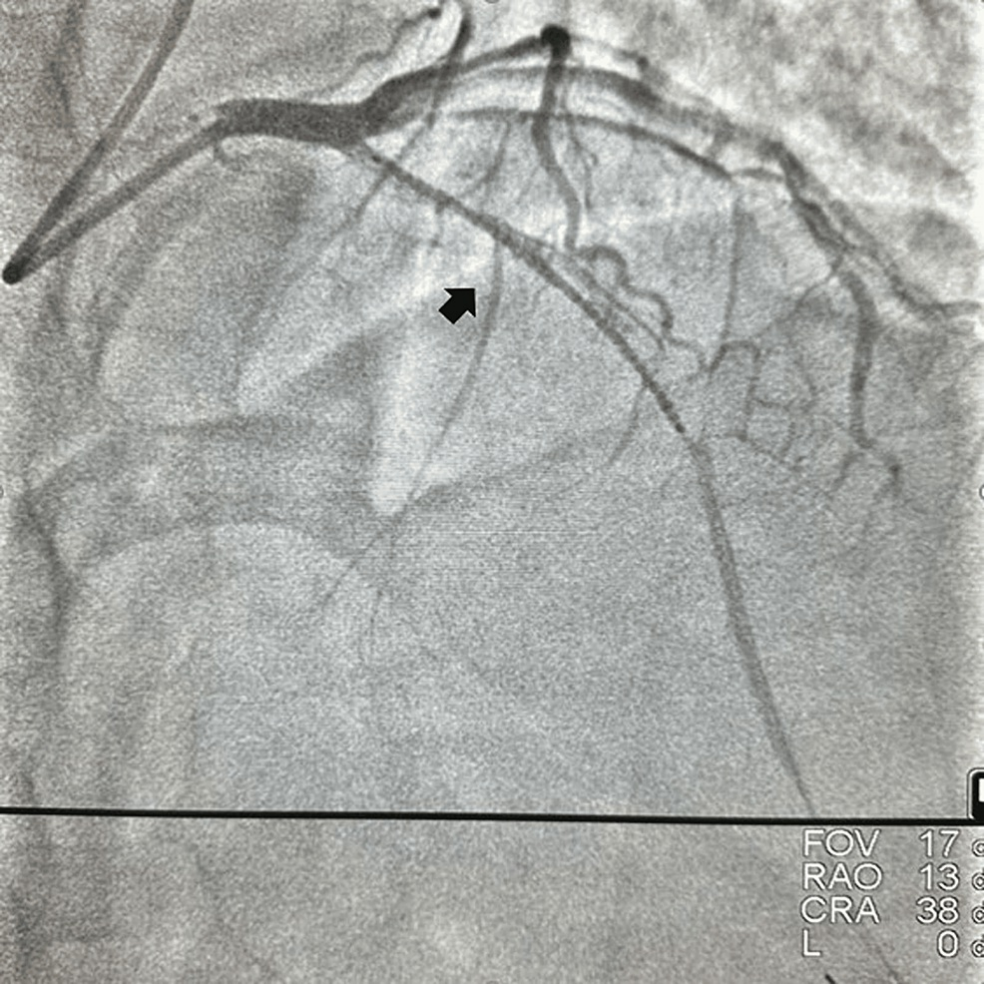
Drug-eluting stent covering the long dissection.

**Figure 5:**
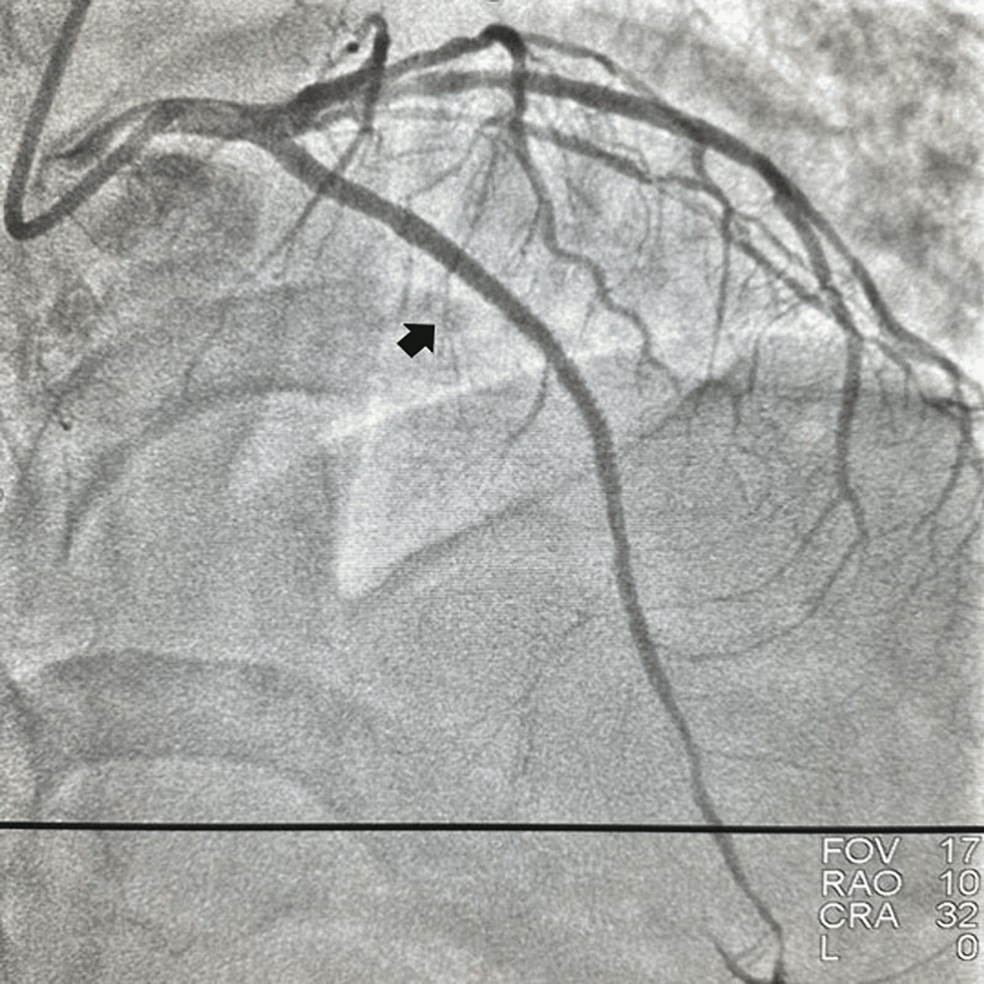
Post-intervention good angiographic result with distal thrombolysis in myocardial infarction III flow.

An 82-year-old diabetic, hypertensive female presented to the emergency department with rest angina Canadian Cardiovascular Society (CCS Class IV) for the last eight hours with diaphoresis and palpitation. She had no history of orthopnea and paroxysmal nocturnal dyspnea in the past. She had exertional angina CCS Class II to III for the last six months and was on baby aspirin 75 mg once daily, atorvastatin 40 mg once daily, metoprolol 50 mg once daily, and nitroglycerine 2.6 mg twice daily. She had a heart rate of 104 beats per minute and a blood pressure of 150/90 mmHg in the right arm supine position with oxygen saturation (SpO_2_) of 98% on room air. An electrocardiogram (EKG) revealed downsloping ST depression with T-wave inversion in anterior precordial leads and troponin was negative. Echocardiography revealed no regional wall motion abnormality with normal ejection fraction (60%) and the presence of degenerative aortic sclerosis. Her serum chemistries were within normal limits. As she had refractory angina despite sublingual nitrate, she was rushed immediately to the Cath lab for injecting the coronaries. A coronary angiogram revealed chronic total occlusion of the osteoproximal left anterior descending coronary artery (LAD) (Figure 1). With a loading dose of ticagrelor and unfractionated heparin of 100 U/kg intravenous, the left main coronary artery was engaged with extra backup 6F 3.5, and the lesion in LAD was tried to cross with Fielder FC, Fielder XT-A wire, respectively, which did not cross the lesion with balloon support. Then the lesion in the osteoproximal LAD was crossed antegradely with Gaia II wire (Figure 2) with balloon support. After the wire entered the distal true lumen, LAD was injected which revealed a long-segment dissection of the left anterior coronary artery (Figure 3) and it was a true lumen-false lumen-true lumen dissection. The patient developed hemodynamic compromise with a blood pressure of 80/40 mmHg for which noradrenaline infusion was started, and she was hypoxic with SpO_2_ of 86% for which she was put on noninvasive ventilation with FiO_2_ of 100% and continuous diuretic infusion. Initially, a long stent of 2.75 mm × 36 mm could not cross the lesion due to the presence of dense calcium. The lesion was further dilated with 2.5 mm × 10 mm and 2.75 mm × 10 mm balloon distally and 3 x 10 mm balloon proximally. After aggressive dilation of the proximal and distal lesions, we negotiated a 3 x 38 mm drug-eluting stent distally and a 3.5 x 44 mm drug-eluting stent proximally (Figure 4) to cover the whole long-segment dissection at 12-14 atm pressure followed by post-dilatation with 3 x 8 mm noncompliant balloon distally and 3.5 x 8mm noncompliant balloon proximally. Post-revascularization with drug-eluting stents, there was a good angiographic result with distal thrombolysis in myocardial infarction III flow (Figure 5). The patient improved hemodynamically with a blood pressure of 110/70 mmHg and hypoxia also improved shortly. She was free from angina and was put on dual antiplatelets including aspirin and ticagrelor, high-dose atorvastatin, nitrate, beta-blocker, and short-term low-molecular-weight heparin in view of the long coronary stent (>50 mm) and preceding complex and difficult intervention. She was discharged in stable condition with fair hemodynamics and an angina-free state on the third day of post-intervention. Intravascular ultrasound imaging of the long-segment coronary dissection could not be done as the patient was hemodynamically unstable with desaturation; additional imaging and spending time would have deteriorated the patient’s condition further.

## Discussion

We present a rare case of a very long segment (>50 mm) coronary dissection during revascularisation of the osteoproximal LAD chronic total occlusion. The incidence of iatrogenic coronary artery dissections is less than 0.1%, but the true incidence is underreported [2,3]. Long-segment coronary artery dissections are less commonly described in the literature. Guide wire-induced dissection is more common in the right coronary artery than in the left coronary artery due to the inherent C-shaped curve of the right coronary artery. Most cases of coronary dissection are managed with stenting (82%), conservatively (12%), and by surgery (6%) [4]. Female sex and complex lesions with high calcium burden were the two most important factors behind this long-segment coronary dissection. The integrity of the antegrade flow is the most important predictor of successful clinical outcome in coronary dissection [5] which was achieved by deploying two long stents inside the coronary artery(>50 mm). The most important factor in preventing a long-segment dissection is the use of a soft workhorse guide wire with a low tip load [6], but the calcific lesion in our case could not be crossed antegradely with a low tip load wire which was ultimately crossed with a Gaia II wire with a high tip load. Intravascular ultrasound provides an adequate assessment of the vessel diameter and length of dissection [7], but we were unable to perform the same so as not to consume more time as the patient was hemodynamically unstable with desaturation. Acute vessel reocclusion occurs in 37% of cases of coronary dissection, but with stenting in the modern era, it has decreased to 2% [8]. In the event of abrupt vessel closure, the use of a 2F microcatheter to aspirate the obstructive hematoma and establish the antegrade flow has been reported [9]. It is important to distinguish dissection in the coronary artery from contrast streaming, overlapping small vessels, and wire-induced severe concentrina. Most iatrogenic coronary artery dissections are limited to the true lumen only but the present case describes a rare guide wire-induced long-segment coronary artery dissection (>50 mm) from the true lumen to the false lumen and again to the true lumen, which successfully revascularized with two long overlapping stents which dramatically improved the hemodynamics, angina scale, and desaturation of the patient.

## Conclusions

We report an interesting and extremely rare guide wire-induced long-segment coronary dissection in the LAD during osteoproximal LAD intervention which was successfully managed by stenting the dissected segment with two long (>30 mm) overlapping stents. Although long-segment coronary dissections from true lumen-false lumen-true lumen are rare and catastrophic, rapid address of the same with long drug-eluting stents can prevent the adverse hemodynamic and clinical consequences of abrupt vessel closure.
